# TeLePhone Respiratory (TeLePoR) score to assess the risk of immediate respiratory support through phone call for acute dyspnoea: a prospective cohort study

**DOI:** 10.1186/s13049-025-01405-3

**Published:** 2025-05-16

**Authors:** Frederic Balen, François Saget, Axel Benhamed, Oussama-Ibrahim Boudjemline, Lisa Girard, Elisa Lescanne, Pauline Mimouni, Paul-Georges Reuter, Sandrine Charpentier, Nicolas Marjanovic

**Affiliations:** 1 Emergency Department, Centre Hospitalier Universitaire de Toulouse, Toulouse, France; 2 CERPOP– EQUITY, INSERM, Toulouse, France; 3 Univ Rennes, CHU Rennes, SMUR / Urgences Adultes, Service SAMU 35, Rennes, France; 4 Emergency Department-SAMU69, Centre Hospitalier Universitaire Edouard-Herriot, Hospices Civils de Lyon, Lyon, France; 5 Département de Médecine d’Urgence, Centre de recherche, CHU de Québec - Université Laval, Québec, Canada; 6 CHU de Poitiers, Service d’Accueil des Urgences et SAMU 86, Poitiers, France; 7 Toulouse III– Paul Sabatier University, Toulouse, 31330 France; 8 IS-ALIVE, INSERM, Poitiers, CIC-1402 France; 9 Faculté de Médecine et de Pharmacie de Poitiers, Université de Poitiers, Poitiers, France

**Keywords:** Dyspnea, Telephone, Emergency medical call centre, Risk

## Abstract

**Background:**

Acute dyspnea is a frequent cause to call the Emergency Medical Call Center (EMCC). The main challenge for EMCC dispatchers is to quickly identify patients that will require respiratory support in order to provide them with the most accurate prehospital response. Our main objective was to derivate a score assessable during the first call to detect the most severe patients needing medical assistance.

**Methods:**

This prospective observational cohort study was conducted in four different French EMCC from January 22nd to March 7th 2024. Patients over the age of 18 years old that called once the EMCC for acute dyspnea were included in our study. The primary endpoint was an immediate respiratory support requirement (i.e. high-flow oxygen, non-invasive ventilation or mechanical ventilation after intubation) before or at the Emergency Department Registration. Variables of interest to predict respiratory support were prospectively collected in each EMCC. A multivariate analysis by stepwise logistic regression was used to select variables associated with the primary endpoint and to create in the TeLePhon Respiratory Score (TeLePoR score). The TeLePoR score was compared to medical dispatcher intuition for predicting respiratory support.

**Results:**

Six hundred and forty-nine patients were analyzed, including 49 (8%) that required immediate respiratory support. The risk factors included in the TeLePoR score were: altered ability to speak complete sentences (OR = 8.62; CI95% = [3.49–21.3]), abdominal respiration (OR = 2.42; CI95% = [1.23–4.76]), altered consciousness (OR = 2.05; CI95% = [0.90–4.65]) and self-report breathing discomfort > 7/10 (OR = 1.83; CI95% = [0.96–3.47]) respectively. Considering these factors, TeLePoR score presented a 0.810 AUC. Medical dispatcher intuition was not statistically superior to TelePoR score to predict immediate respiratory support (AUC = 0.836 vs. 0.810; *p* = 0.431).

**Conclusion:**

TeLePoR score is a simple scoring system including 4 variables to predict immediate respiratory support in patients calling the EMCC for acute dyspnea.

**Supplementary Information:**

The online version contains supplementary material available at 10.1186/s13049-025-01405-3.

## Introduction

Acute dyspnea is a frequent reason for contacting the Emergency Medical Call Center (EMCC), representing over 8% all calls [1]. The most severe patients, presenting acute respiratory distress syndrome require respiratory support (such as non-invasive ventilation (NIV)) in prehospital settings in order to reduce their morbimortality [2]). NIV or other respiratory supports (i.e. high-flow oxygen or mechanical ventilation after intubation) should be initiated by a specialized team led by a physician or a specialized paramedic, in prehospital settings. These teams are also trained at administering appropriate medication for dyspnea [3, 4]. However, logistical limitations preclude the deployment of such teams to all patients reporting dyspnea, as only approximately 15% of those transported to a hospital subsequently require early respiratory support [5].

Evidence suggests that immediate dispatch of specialized teams following the initial EMCC call correlates with improved clinical outcomes in the most critically ill patients, compared to delayed response time [6]. Additionally, EMCC dispatchers’ main challenge is to quickly identify patients that will require respiratory support in order to adapt the most accurate prehospital response. While several studies have demonstrated that vital parameters measurable by ambulance staff (e.g., respiratory rate or oxygen saturation) are useful indicators to identify high-risk dyspneic patients [7, 8], the investigation about risk factors that can be reliably assessed during a phone consultation remains limited [9].

In a retrospective cohort study that we had previously published, we identified 6 risk factors of early respiratory support assessable when the EMCC was contacted for the first time regarding dyspnea: β2-mimetics as usual treatment, polypnea, altered ability to pronounce complete sentences, cyanosis, sweats and altered consciousness [5]. This study presented, nevertheless, several limitations. Firstly, due to its retrospective design, at the beginning we were unable to assess some potential interesting predictors (such as abdominal respiration or self-report breathing discomfort [10]). Secondly, we predicted an *early* respiratory support (i.e.in prehospital setting or during within the 3 h after hospital registration). However, expecting an *immediate* respiratory support (i.e. in prehospital setting or during within the first hour after hospital registration) seems relevant for clinical practice. Finally, in our previous retrospective work, we had not compared our model to any other standard practice (medical dispatcher intuition).

Also, the main objective of this prospective cohort study was to derivate a score to predict immediate respiratory support assessable during the first call to EMCC for dyspnea. Our secondary objective was to compare this score to the medical dispatcher intuition in the prediction of immediate respiratory support.

## Methods

### Study design and settings

This prospective observational cohort study was conducted across four EMCC in France from January 22nd to March 7th 2024. In the French EMCC system, incoming calls are initially managed by a call taker who records the caller’s identity and the reason for assistance. If dyspnea is identified as the chief complaint, the call will be transferred to an emergency physician for medical evaluation and regulation. He or she will conduct a thorough medical interview to assess the patient’s condition and determine the appropriate intervention, which may include either medical advice or first-aid team dispatch. This team may include an basic life-support ambulance, or a Mobile Intensive Care Unit (MICU) staffed with a physician and nurse, depending on the patient’s severity. The decision-making process regarding the dispatch of these units is not protocolized and varies across all EMCC. The decision is based on the physician’s clinical discernment to decide whether to dispatch a MICU if respiratory distress is identified during the call. Following the initial deployment of the first-aid team, the EMCC subsequently coordinates with hospitals to settle the patient’s transfer, taking into account both the patient’s medical requirements and the current capacity of nearby healthcare facilities [11]. In France, MICU are the only prehospital team able to provide respiratory support such as high-flow oxygen, NIV, manual and mechanical ventilation after intubation. Patients transported by paramedics without MICU will benefit from such support at the ED arrival if necessary.

### Participants

Patients over the age of 18 years that called the EMCC once for acute dyspnea (< 7 days) were included. Exclusion criteria were: cardiac arrest during the initial call, patients identified as not-to-be-resuscitated, special circumstances (i.e. traumatism or anaphylaxis) and patients that refused to participate. This study has been registered by the University Hospital of Toulouse in line with the French MR-004 Methodology (CNIL number: 2206723 v 0; Institutional Register Number: RnIPH 2023-87).

### Primary end-point and variables

The primary endpoint was an immediate respiratory support (i.e. high-flow oxygen, NIV, manual or mechanical ventilation after intubation) prior to hospital registration (initiated by MICU) or at ED registration (within the first hour after registration). Patients that died before arriving to the hospital were also considered as presenting the primary endpoint.

Variables of interest to predict respiratory support were prospectively collected in the EMCC by the Emergency Physician (EP) during the first medical interview. During this call, EP were requested to collect patients’ usual treatment (especially furosemide and β2-mimetics), dyspnea duration before call, if patients presented tachypnoea, abnormal respiratory noises, inability to speak full sentences, cyanosis, sweats, abdominal respiration or altered consciousness [5]. If possible, self-report breathing discomfort was collected using a numerical scale from 0 (no discomfort) to 10 (worst breathing discomfort imaginable) [10]. The EP intuition on the risk of immediate respiratory support was also collected on a scale from 0 (no risk) to 10 (the patient is very likely to need respiratory support immediately). Parameters at first contact, pathway after ED, 7-day mortality and final diagnosis after discharge were also collected from hospital charts if possible (i.e. if patients had/presented a first contact with at least an ambulance and eventually was taken to a hospital).

### Study size

Based on findings from a preliminary study [5], we hypothesised that 10 to 15% of patients would require immediate respiratory support. With 1000 patients, we could have identified between 100 and 150 that may have required immediate respiratory support. We initially planned to split the cohort in two groups for development (2/3) and internal score validation (1/3). This number of inclusions should allow us to explore between 7 and 10 potential predictors in the derivation cohort, considering that multivariate analysis requires 10 events per variable included in the model. During the 1.5 month of inclusion, we managed to include 652 patients. In the absence of fundings, the study could not be extended any further. Conservatively, we finally developed one predictive score with no internal validation.

### Statistical analyses

Data were analysed with STATA software (version 16; StataCorp, College Station, TX). No imputation was used in order to describe the population. Quantitative variables were described with median and IQR (m (q1-q3)) and Mann-Whitney test was used to compare groups. The threshold for self-report breathing discomfort was chosen using Liu cut-point method to maximise both sensitivity and specificity. Qualitative variables were described with number and percentage (n(%)). Moreover, Chi2 or exact-Fischer test was used to compare groups. To identify predictive factors of immediate respiratory support, we used stepwise logistic regression. Missing values regarding potential predictors were considered as normal. The final multivariate analysis only showed risk factors remaining associated with p-value < 0.05. A scoring system was therefore developed (the TeLePhon Respiratory Score (or Toulouse-Lyon-Poitiers-Renne Score) (TeLePoR Score), based on the risk factors identified. The test characteristics (i.e. sensitivity, specificity, positive and negative predicting values) of TeLePoR score were also calculated with their 95% confidence interval (95%CI) for every threshold. Afterwards, the score performance was compared to medical dispatcher’s intuition.

## Results

### Patient characteristics

A total of 656 patients were screened for inclusion. After excluding 7 patients (2 refused to participate, 2 double inclusions, 3 non-related dyspnea calls), 649 patients were analyzed, of whom 49 (8%) required immediate respiratory support. Patients’ characteristics at call and final decision of EMCC dispatch are depicted in Table 1. Initial vital parameters and patients’ final diagnosis that required either an ambulance or ED visit are represented in Table 2. NIV was the most frequent respiratory support required (*n* = 41 (84%)) (Table 2). Most frequent diagnoses at discharge were bacterial pneumonia (143 (26%)), acute heart failure (111 (20%)), and COPD exacerbation (75 (14%)), respectively.

**Table 1:** Population’s characteristics assessed via telephone call

	Population	No respiratory support	Respiratory support required	*p*-value
	(*n* = 649)	(*n* = 600)	(*n* = 49)	
Age (years old)	77 (65 - 87)	78 (64 - 87)	72 (65 - 78)	**0.04**
Women	366 (56)	341 (57)	25 (51)	0.430
Medical history:				
- Heart disease	344 (53)	316 (53)	28 (57)	0.546
- Lung disease	307 (47)	277 (46)	30 (61)	**0.042**
- Chronic renal failure	51 (8)	47 (8)	4 (8)	0.934
- Diabetes	107 (17)	96 (16)	11 (23)	0.242
- Dementia	49 (8)	46 (8)	3 (6)	0.694
Usual treatment:				
- Furosemide	182 (28)	172 (29)	10 (20)	0.216
- B2-mimetics	179 (28)	164 (27)	15 (31)	0.621
Duration of symptoms before call (hours)	13 (2 - 57)	13 (2 - 57)	5 (1 - 23)	**0.014**
Duration of symptoms ≥ 5 h	406 (63)	382 (64)	24 (50)	**0.041**
Tachypnea:	407 (63)	365 (61)	42 (86)	**0.001**
- Not evaluated	36 (6)	34 (6)	2 (4)	1
Abnormal respiratory noises:	316 (49)	284 (47)	32 (65)	**0.005**
- Wheezing	135 (21)	123 (21)	12 (25)	**0.008**
- Crackling	177 (27)	158 (26)	19 (40)	
- No	308 (47)	295 (49)	13 (27)	
- Not evaluated	29 (5)	24 (4)	5 (10)	
Unable to speak:	249 (38)	206 (34)	43 (88)	**< 0.001**
- Not evaluated	34 (5)	34 (6)	0	0.100
Cyanosis:	90 (14)	79 (13)	11 (22)	**0.071**
- Not evaluated	44 (7)	38 (6)	6 (12)	0.132
Sweats:	104 (16)	90 (15)	14 (29)	**0.013**
- Not evaluated	60 (9)	54 (9)	6 (12)	0.440
Abdominal respiration:	223 (34)	189 (32)	34 (69)	**< 0.001**
- Not evaluated	120 (18)	111 (19)	9 (18)	1
Altered consciousness:	56 (9)	46 (8)	10 (20)	**0.002**
- Not evaluated	10 (2)	10 (2)	0	1
Breathing discomfort (0 to 10)	7 (5 - 8)	7 (5 - 8)	8 (7 - 10)	**< 0.001**
- Breathing discomfort > 7	178 (27)	153 (26)	25 (51)	**< 0.001**
- Not evaluated	168 (26)	152 (25)	14 (29)	0.869
Medical dispatcher intuition (0 to 10)	2 (1 - 5)	2 (1 - 4)	7 (5 - 8)	**< 0.001**
EMCC final decision:				
- Medical advise or GP alone	104 (16)	103 (17)	1 (2)	N.A
- Dispatch of an ambulance alone	463 (71)	448 (75)	15 (31)	
- Dispatch of a MICU after ambulance assesment	30 (5)	14 (2)	16 (33)	
- Dispatch of a MICU immediatly after call	52 (8)	35 (6)	17 (35)	

**Table 2:** Population’s characteristics at first contact, final diagnosis and pathway

	Patients with one contact other than GP	No respiratory support	Respiratory support required
	(*n* = 546)	(*n* = 497)	(*n* = 49)
Parameters at first contact:			
- Respiratory rate > 22 cpm	259 (47)	231 (46)	28 (57)
- SpO2 < 90%	239 (44)	201 (40)	38 (78)
- GLS ≤ 14	32 (6)	17 (3)	15 (31)
- SBP < 90 mmHg	18 (3)	14 (3)	4 (8)
- HR > 100 bpm	174 (32)	151 (30)	23 (47)
Respiratory support required:			
- High flow oxygen	2 (<1)	-	2 (4)
- Non-invasive ventilation	41 (8)	-	41 (84)
- Mechanical ventilation	4 (1)	-	4 (8)
- Prehospital death w/o support	2 (<1)	-	2 (4)
Final diagnosis:			
- More than one diagnosis	51 (9)	47 (9)	4 (8)
- Acute heat failure	111 (20)	96 (19)	15 (31)
- Bacterial pneumonia	143 (26)	130 (26)	13 (27)
- COPD exacerbation	75 (14)	61 (12)	14 (29)
- Acute asthma	13 (2)	13 (3)	0
- Pulmonary embolism	12 (2)	11 (2)	1 (2)
- Viral infection	68 (12)	63 (13)	5 (10)
- Others	73 (13)	67 (13)	6 (12)
- Unknown	172 (32)	168 (34)	4 (8)
Death before hospital admission	4 (1)	0	4 (8)
Patients attending the hospital	484 (89)	439 (88)	45 (92)
Hospital admission after ED	302 (55)	257 (52)	45 (92)
7 day-mortality	40 (7)	27 (5)	13 (27)

SaO2: Oxygen saturation level; GLS: Glasgow Score; GP: General Practitioner; SBP: Systolic Blood Pressure; HR: Heart Rate

### Main results

**Fig. 1:**
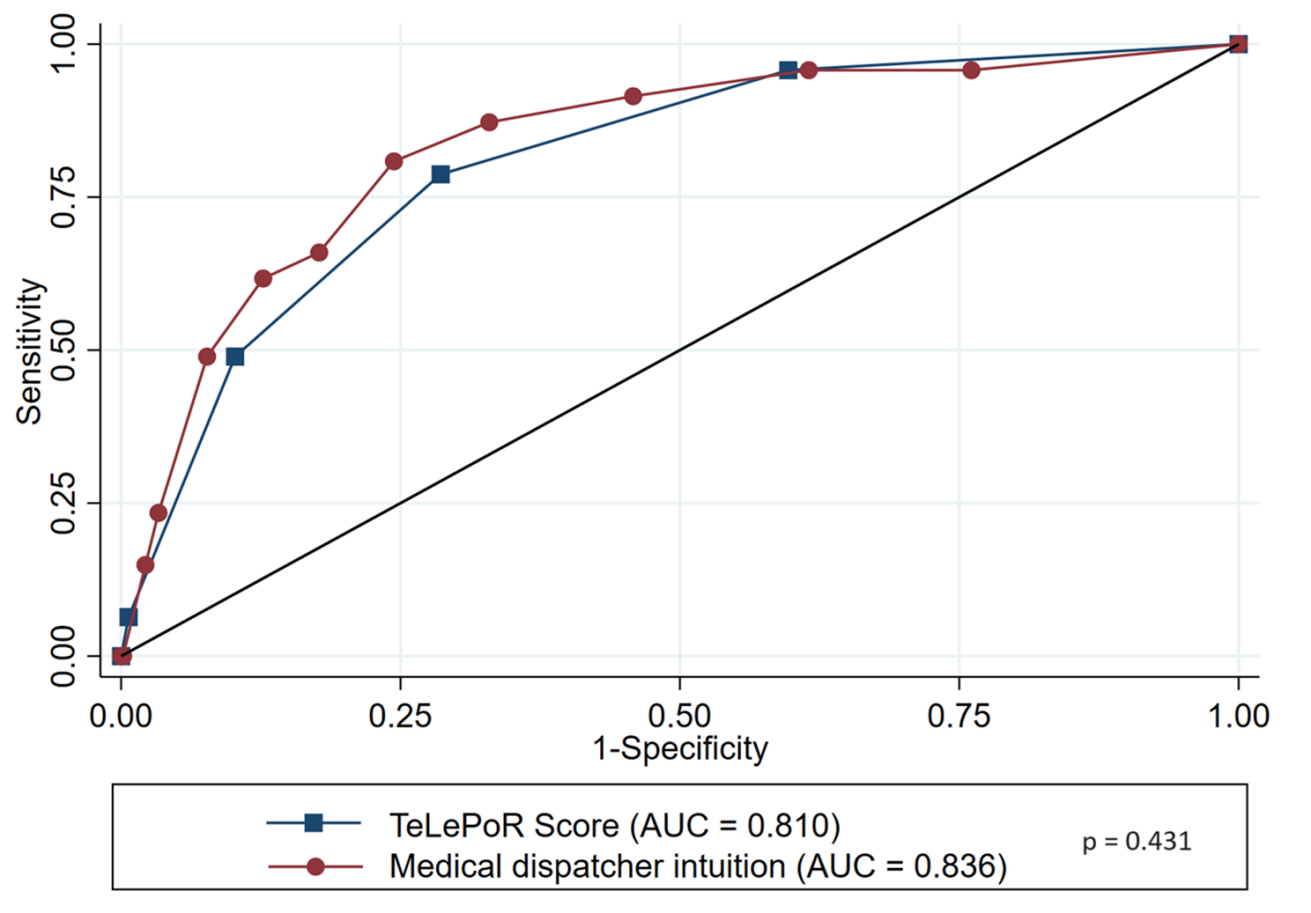
ROC of TeLePoR score and Medical dispatcher intuition in prediction of respiratory support requirement

Predictive factors independently associated with immediate respiratory support with p-value < 0.05 in multivariate analysis were: altered ability to speak complete sentences (OR = 8.62; CI95% = [3.49–21.3]), abdominal respiration (OR = 2.42; CI95% = [1.23–4.76]), altered consciousness (OR = 2.05; CI95% = [0.90–4.65]) and self-report breathing discomfort > 7/10 (OR = 1.83; CI95% = [0.96–3.47]) (Table 3). TeLePoR Score presented a 0.810 AUC (Table 4). Two hundred and twenty-four (38%) patients showed no predictive risk factors, leading to a 0% risk of immediate respiratory support. Patients with 3 (*n* = 77 (12%)) and 4 (*n* = 7 (1%)) risk factors had a high (26%) and very high (43%) risk to require immediate respiratory support, respectively. The test characteristics of TeLePoR score are shown in Table 5. Medical dispatcher’s intuition was not statistically superior to TelePoR score to predict immediate respiratory support (AUC = 0.836 vs. 0.810; *p* = 0.431) (Fig. 1).

**Table 3:** Predictive factors at call of immediate respiratory support

	OR	[CI95]
Altered ability to speak complete sentences	8.62	[3.49 - 21.3]
Abdominal respiration	2.42	[1.23 - 4.76]
Altered consciousness	2.05	[0.90 - 4.65]
Self report breathing discomfort > 7 /10	1.83	[0.96 - 3.47]

**Table 4:** Risk of immediate respiratory support according to telepor score (AUC = 0.810)

Points (number of risk factors)	Patients (*n* (%))	Respiratory support (*n*)	Risk (%; [95CI])	Risk class
0	244 (38)	2	1% [0 - 3]	Very Low
1	195 (30)	8	4% [2 - 8]	Low
2	126 (19)	16	13% [7 - 20]	Intermediate
3	77 (12)	20	26% [17 - 37]	High
4	7 (1)	3	43% [9 - 82]	Very High
Total	649	49	8% [6 - 10]	-

**Table 5:** Performances of telepor score in prediction of immediate respiratory support requirement

	Sensitivity (% [95CI])	Specificity (% [95CI])	PPV (% [95CI])	NPV (% [95CI])
- < 1 point	96 [86 - 100]	40 [36 - 44]	12 [8 - 15]	99 [97 - 100]
- < 2 points	80 [66 - 90]	72 [68 - 75]	19 [14 - 25]	98 [96 - 99]
- < 3 points	47 [33 - 62]	90 [87 - 92]	27 [18 - 38]	95 [93 - 97]
- < 4 points	6 [1 - 17]	99 [98 - 100]	43 [91 - 95]	93 [91 - 95]

PPV and NPV: Positive and Negative Predictive values

## Discussion

We identified four risk factors assessable via phone-call to the EMCC for dyspnea in order to predict immediate respiratory support such as: altered ability to speak complete sentences, altered consciousness, abdominal respiration, and self-report breathing discomfort > 7/10. The impossibility of speaking full sentences had been previously described in literature as an indicator of severe dyspnea. Indeed, the *Roth* score (patients’ ability to count to 30 in one breath) was described to predict SpO2 < 95% [12]. Altered consciousness is a sign associated with hypercapnia being an NIV indication in respiratory failure [13]. Other previously described risk factors (i.e. polypnea, cyanosis, sweets and β2-mimetics as usual treatment [5]) do not remain in the final model suggested herein. This might be explained by two hypotheses; first of all, the outcome was not evaluated at the same time in this paper. Indeed, *immediate* respiratory support had been evaluated prior to hospital admission within the first hour after admission compared to our previous study in which *early* respiratory support evaluated before or during the 3 h after admission. Secondly, other risk factors that had not been explored in our previous study were probably strongly correlated with our main outcome (see *Supplementary File*). Furthermore, abdominal paradoxical breathing is an important sign that defines respiratory distress [14]. Self-report breathing discomfort has been recently described as relevant information to predict death and hospital resources applied in prehospital [8] or hospitalized patients [10]. Moreover, patients feel differently regarding dyspnea [15], therefore, it is key to request them how they feel and their discomfort degree during the interrogatory. Stevens JP et al. [10] proposed a threshold of 4 on a scale of 10 to predict hospital bad outcomes in their population. In our cohort, a threshold of 7 out of to 10 is likely to be more relevant to predict immediate respiratory support.

Our study population is comparable to previous studies about ambulance transport of dyspneic patients [8, 16]. Above half of the patients required hospital admission after attending the ED and the 7-day mortality was 7%. The most frequent final diagnoses were bacterial pneumonia, acute heart failure and COPD exacerbation which are frequent in ED settings [17, 18]. Immediate respiratory support rate was 8% in our study, which is consistent with the 15% rate of respiratory support during the 3 h after admission [5].

The TeLePhon Respiratory score (or Toulouse-Lyon-Poitiers-Renne score) (TeLePoR score) that we propose seems equivalent to medical dispatcher’s intuition to predict immediate respiratory support. However, medical dispatcher’s intuition was gathered after collecting the variables of interest during the first phone call. It is also impossible to know if the medical dispatcher would have provided comparable results without any guided interrogatory. Moreover, the four risk factors that compose the TeLePoR score are probably assessable by a non-physician call-taker. While telephone interviews in French EMCC are usually led by emergency physicians [11], it remains uncommon in other EMCC worldwide. Furthermore, it is probably interesting to easily identify the most severe patients calling non-medical staffed EMCC for dyspnea. The TeLePoR score use may guide dispatch of advanced-life support teams (i.e. MICU in France, or paramedic trained teams in other countries [3]). In our cohort, patients that showed 0 TeLePoR score (38% of the cohort) or 1 (30% of the cohort) had very low (0%) or low (4%) risk of immediate respiratory support. They probably do not require immediate dispatch of an advanced-life support team. Patients that presented a 3 TelePoR score (12% of the cohort) and 4 (1% of the cohort) had a high (26%) and very high (43%) risk of requiring immediate respiratory support. Those patients might need immediate dispatch of an advanced-life support team. Looking at our cohort, this suggested strategy would have increased the number of immediate dispatches of advanced-life support team from 8 to 13% in order to promptly detect 34% vs. 47% of patients that required immediate respiratory support. This might be interesting for patients’ outcomes, considering that delayed advanced life support team dispatch compared to immediate dispatch for respiratory distress patients is harmful [6]. In the future, a randomised trial to compare standard vs. TeLePoR-guided dispatch will be necessary to ensure the relevance of this score in patients’ outcomes and improve medico-economic aspects.

### Limitations

The main limitation of our study is that the reproducibility of variables of interest between potential operators (call takers) has not been assessed. This reproducibility also depends on the person who calls (the patient himself or a witness). This is probably more problematic for abdominal respiration that might be easier to evaluate for a witness than for the patient himself. Moreover, relevant clinical signs (abdominal breathing, cyanosis, consciousness…) will be probably easier to evaluate in the future through telemedicine as video-call [19]. Such tool should lead to improve TeLePoR score assessment, but it will also require being inter-operator validated. Another limitation of our study consists in the fact that only one score has been developed, but it has not been internally validated yet due to a lack of patients enrolled in the study. Moreover, our score requires at least one external validation study. A prospective external validation proposal in a non-French setting would be particularly relevant in order to study our model with a non-physician staffed EMCC. Finally, the medical dispatcher’s intuition was assessed after EP’s collected variables of interest through phone call. This may artificially improve EP’s intuition performances by guiding it.

## Conclusion

Altered ability to speak complete sentences, altered consciousness, abdominal breathing, and self-report breathing discomfort > 7/10 are independent risk factors of immediate respiratory support assessable via phone-call to the EMCC for dyspnea. Considering those risk factors, we have settled the TeLePoR score. TeLePoR score performance is not superior to Medical dispatcher’s intuition.

## Electronic supplementary material

Below is the link to the electronic supplementary material.

Supplementary Material 1

## Data Availability

Data are available upon reasonable request to the corresponding author.
